# Aged Mice are More Resistant to Influenza Virus Infection due to Reduced Inflammation and Lung Pathology

**DOI:** 10.14336/AD.2017.0701

**Published:** 2018-06-01

**Authors:** Jiao Lu, Xuefeng Duan, Wenming Zhao, Jing Wang, Haoyu Wang, Kai Zhou, Min Fang

**Affiliations:** ^1^CAS Key Laboratory of Pathogenic Microbiology and Immunology, Institute of Microbiology, Chinese Academy of Sciences, Beijing 100101, China.; ^2^University of Chinese Academy of Sciences, Beijing, China; ^3^Institute of Health Sciences, Anhui University, Hefei, China; ^4^International College, University of Chinese Academy of Sciences, Beijing, China

**Keywords:** ageing, immunosenescence, cellular immunology, IAV infection, inflammation, lung pathology

## Abstract

Immune responses are a double-edged sword. Effective and appropriate immune responses capable of controlling viral infection while also largely preserving tissue integrity, are critical for host survival. Too strong immune responses might result in immune pathology, while too weak immune responses might cause viral persistence. Physiologic ageing is accompanied with a decline in the normal functioning of the immune system, which is termed as "immunosenescence". We show that aged mice (16-19 months old) are more resistant to influenza A virus (IAV) infection than the young mice. Strong immune responses in the young mice after IAV infection result in faster clearance of virus, but also cause severe lung injury and higher mortality rate. While in the aged mice, the delayed and milder immune responses contribute to reduced pulmonary damage, and are still capable to clear the infection even with a slower kinetics, displaying a more resistant phenotype during IAV infection. Hence, our work demonstrates that moderate immune responses as a decline with ageing in the aged mice balance the immune pathology and viral clearance, might be beneficial for the host during certain circumstances. Our results provide important insight to our basic knowledge of immunosenescence and immune defenses to invading pathogens. Further, our results indicate that age factors should be considered when investigating the vaccination and therapeutic strategies for severe IAV infection.

The global population is rapidly aging. The share of people aged 60 years and older in the next four decades is expected to rise to 22% of the total population-a jump from 800 million to 2 billion people [1]. The ageing of populations is becoming the next global public health challenge [2, 3]. Aging accompanied with the progressive decline in immune function, termed “immuno-senescence”. Immunosenescence includes a defective adaptive response and changes of the innate immune system [4-6]. Immunosenescence contributes to reduced efficacy to vaccination and increased susceptibility to infectious diseases in the elderly [7, 8]. Several human diseases including arthritis, cancer, cardiovascular, diabetes, neurodegenerative, bacterial and viral infections are closely associated with ageing and inflammation [9, 10].

Influenza virus belongs to the orthomyxovirus family and are divided into four types, including types A, B, C and D (http://www.cdc.gov/flu/about/viruses/types.htm) [11]. Type A and B viruses are important causes of diseases in human, in which the majority of cases are due to type A virus [12]. Worldwide, the IAV infections are estimated to result in about 3 to 5 million cases of severe illness, and about 250 000 to 500 000 deaths annually (http://www.who.int/mediacentre/factsheets/fs211/en/). Influenza-associated morbidity and mortality disproportionately affect older adults [13]. It has been reported that ≥90% of annual influenza-related deaths occur in individuals ≥65 years of age [14, 15].

Induction of both innate and adaptive immune responses is crucial for the control of viremia after IAV infection. However, the current understandings of IAV infection suggest that excessive host-immune responses lead to immunopathology followed by respiratory dysfunction and mortality[16, 17]. After IAV infection, pro-inflammatory responses are initiated by the host and involve the production of pro-inflammatory cytokines (IFN-α, IFN-γ, TNF-α and IL-6) and chemokines (KC, MIP-1α, MIP-2, CCL2, CCL5, CXCL10) which facilitate the initial phase of innate immune cell recruitment into the lungs [18, 19]. Although pro-inflammatory responses are critical for the early control of viral replication, excessive inflammation also increases the level of tissue damaging cytotoxic and pro-apoptotic products in the lungs, which result in severe complications leading to death [20, 21]. During highly pathogenic viral infections, including influenza A virus H1N1 [22], H5N1 [23], H7N9 [24], severe acute respiratory syndrome coronavirus (SARS-Cov) [25], Middle East respiratory syndrome coronavirus (MERS-Cov) [26], the immune inflammation and hypercytokinemia, or “cytokine storm,” induce severe lung injury, which is the most common cause of death in patients [27]. In the elderly population, due to the decline in the normal functioning of the immune system, the ability to initiate primary immune response against novel antigens decreases with age [6, 28, 29]. And this may benefit for the elderly to infections in some cases. For example, during the early weeks of the 2009 pandemic of influenza A/H1N1 (pH1N1) in Mexico, the majority of people who died of pneumonia were under 50 [30]. Comparison of patients hospitalized in China with IAV showed that the median age of individuals confirmed infection with H7N9 was 63 years old, while with H5N1 infection was 26 years old and with 2009 pandemic H1N1 infection was 25 years old [31, 32]. These reports indicate that different influenza viruses might have distinct age patterns of severe infection and symptom. However, many factors influence the final outcome after IAV infection. Factors like cross-reactive antibodies in the case of the 2009 pandemics and economic activities on avian influenza infection might play dominant roles for the patterns during some infections [16, 30, 33].

Here we demonstrated that the aged mice were more resistant to H1N1 PR8 infection than the young mice. Reduced immune-pathological reactions in the aged mice after influenza virus infection might be an important reason for the increased survival in the aged mice.

## MATERIALS AND METHODS

### Ethics statement

The mouse experimental design and protocols used in this study were approved by the Regulation of the Institute of Microbiology, Chinese Academy of Sciences of Research Ethics Committee (permit no. PZIMCAS2012008). All mouse experimental procedures were performed in accordance with the Regulations for the Administration of Affairs Concerning Experimental Animals approved by the State Council of People’s Republic of China.

### Cells

The B6-derived dendritic cell (DC) line DC2.4 [17] was a gift from Dr. K. Rock (University of Massachussetts Medical Center, Worcester, MA). Madin-Darby canine kidney (MDCK) cells (ATCC, CCL-34) were purchased from ATCC, and cultured with complete DMEM medium. As standard tissue culture medium, we used complete RPMI or DMEM which consisted of the indicated tissue culture medium supplemented with 10% FCS, 100 IU/ml penicillin, 100 μg/ml streptomycin, 10 mM HEPES buffer (all from Gibco), and 0.05 mM 2-ME (Amresco). All cells were grown at 37°C and 5% CO_2_.

### Virus, mice and infections

The mouse-adapted influenza A/Puerto Rico/8/34 (H1N1; PR8) strain was propagated in the chorio-allantoic cavities of 10-day-old specific pathogen-free (SPF) embryonated chicken eggs (Beijing Merial Vital Laboratory Animal Technology) for 48-72 h at 37° C. Allantoic fluids were then harvested and stored in aliquots at -80° C. Virus titers were determined by plaque assays on MDCK cells as previously described [34]. Briefly, 10-fold serial dilutions of the virus were used to infect confluent MDCK cells in 12-well plates for 1 h at 37° C. The virus inocula were removed by washing with PBS. Cell monolayers were overlaid with agar medium (DMEM supplemented with 1% low melting point agarose and 1 μg/ml N-tosyl-L-phenylalanyl chloro-methyl ketone-treated trypsin) and incubated at 37° C for 48-72 h. The plates were fixed with 4 % paraformaldehyde for 1 h and then the agarose overlays were carefully removed. Staining buffer (0.1 % crystal violet and 20 % ethanol in water) was added to the wells and incubated for at least 10 min. The staining buffer was subsequently aspirated, the plaques counted and the virus titers calculated accordingly.

Female C57BL/6 mice (B6) were purchased from Vital River, China. All mice were housed in an animal facility under specific pathogen-free conditions. For infection experiments, mice were transferred to a biosafety level 2 room. The young mice (7-9 weeks of age) and aged mice (16-19 months of age) were intraperitoneally (i.p.) anaesthetized with trichloro-acetaldehyde hydrate (375 mg/kg body weight) and inoculated intranasally (i.n.) with 1×10^3^ pfu PR8 in 20 μl sterile PBS. Control mice were given an equal volume of PBS i.n. for mock infection. Following infection, weight loss and survival of the infected mice was observed daily. In addition to mice that were found dead, mice with weight loss >25% of the original body weight were euthanized and recorded as dead.

### Isolation of lung lymphocytes

The isolation of lung lymphocytes was adapted as described previously[35]. Briefly, mice were exsanguinated from the orbital cavity to decrease the amount of blood in the lung. The lung was then removed and passed through a cell strainer (BD Falcon) to obtain a single-cell suspension. The cells were resuspended in 35% Percoll solution (in D-Hank’s buffer) and centrifuged at 830 x g for 15 min at room temperature. The upper liquid phase was removed from the tube; the lymphocyte pellet was resuspended in 0.84% NH4Cl solution to lyse the RBCs and then washed twice with PBS containing 2 % FBS. Cells were washed and resuspended in complete RPMI medium.

### Flow cytometric analysis

Detection of NK cell responses was performed as previously described[35]. In brief, to determine NK cell responses in the lung, 2×10^6^ cells were incubated in a 96-well plate at 37° C in complete RPMI medium containing 50 IU/ml IL-2, monensin (BD Biosciences), and FITC conjugated anti-CD107a Ab for 2 h. Then brefeldin A (Sigma-Aldrich) was added to the wells and incubated for another 2 h. The cells were then stained with surface molecules, fixed, permeabilized, and stained for intracellular cytokines using the Cytofix/Cytoperm kit according to the manufacturer’s instructions. Detection of the alveolar macrophages, neutrophils and DC cells was immediately performed by surface staining [36, 37].

To determine T cell responses in lung, 2×10^6^ lymphocytes were cultured at 37°C in 96-well plates in the presence of 2×10^5^ PR8-infected DC2.4 cells or uninfected DC2.4 cells as control. After 5 h, brefeldin A was added to block the secretory pathway and allow for the accumulation of IFN-γ inside the cells. 2 h later, the cells were then stained with surface molecules, fixed, permeabilized, and stained for intracellular cytokines as before.

The following antibodies were used: anti-CD3 (145-2C11), anti-CD4 (GK1.5), anti-NK1.1 (PK136), anti-CD8 (53-6.7), anti-IFN-γ (XMG1.2), anti- CD107a (1D4B; BD Biosciences), and anti-CD11c (N418, all from Sungene Biotech); anti-Gr1 (RB6-8C5), anti-F4/80 (BM8, eBioscience); and anti-CD11b (M1/70, BD Pharmingen). Stained cells were analysed with a FACS Calibur flow cytometer (BD Biosciences). Data were analyzed with FlowJo software (Tree Star).

### Quantitative real-time PCR

**Table 1:** Oligonucleotides and probes used for real-time qPCR.

Gene	Forward Oligonucleotide	Reverse Oligonucleotide	Probe #
Ifnb1	5′-ctggcttccatcatgaacaa-3′	5′-agagggctgtggtggagaa-3′	18
Ifna4	5′-tcaagccatccttgtgctaa-3′	5′-gtcttttgatgtgaagaggttcaa-3′	3
Ifna-non4	5′-ARSYtgtStgatgcaRcaggt-3′	5′-ggWacacagtgatcctgtgg-3′	76
Ifng	5′-atctggaggaactggcaaaa-3′	5′-ttcaagacttcaaagagtctgaggta-3′	21
Il6	5′-gctaccaaactggatataatcagga-3′	5′-ccaggtagctatggtactccagaa-3′	6
Tnfa	5′-tcttctcattcctgcttgtgg-3′	5′-ggtctgggccatagaactga-3′	49
Gapdh	5′-tgtccgtcgtggatctgac-3′	5′-cctgcttcaccaccttcttg-3′	80

Universal Probe Library probes were purchased from Roche. Primers for cytokines were synthesized at Sangon Biotech, beijing. The primers used are listed in Table 1. Total RNA was extracted from lungs of infected mice with TRIzol (Invitrogen). First-strand cDNA was synthesized using oligo-dT primers. Quantitative real-time PCR was performed using a LightCycler 480 (Roche). The cycling conditions for RT-PCR were: 95° C for 10 min, followed by 40 cycles of 95° C for 10 s, 60° C for 30 s and 72° C for 1 s. The fold increase in mRNA expression was determined using the ∆∆Ct method relative to the values in the mock-treated samples after normalization to GAPDH gene expression.

**Figure 1.**
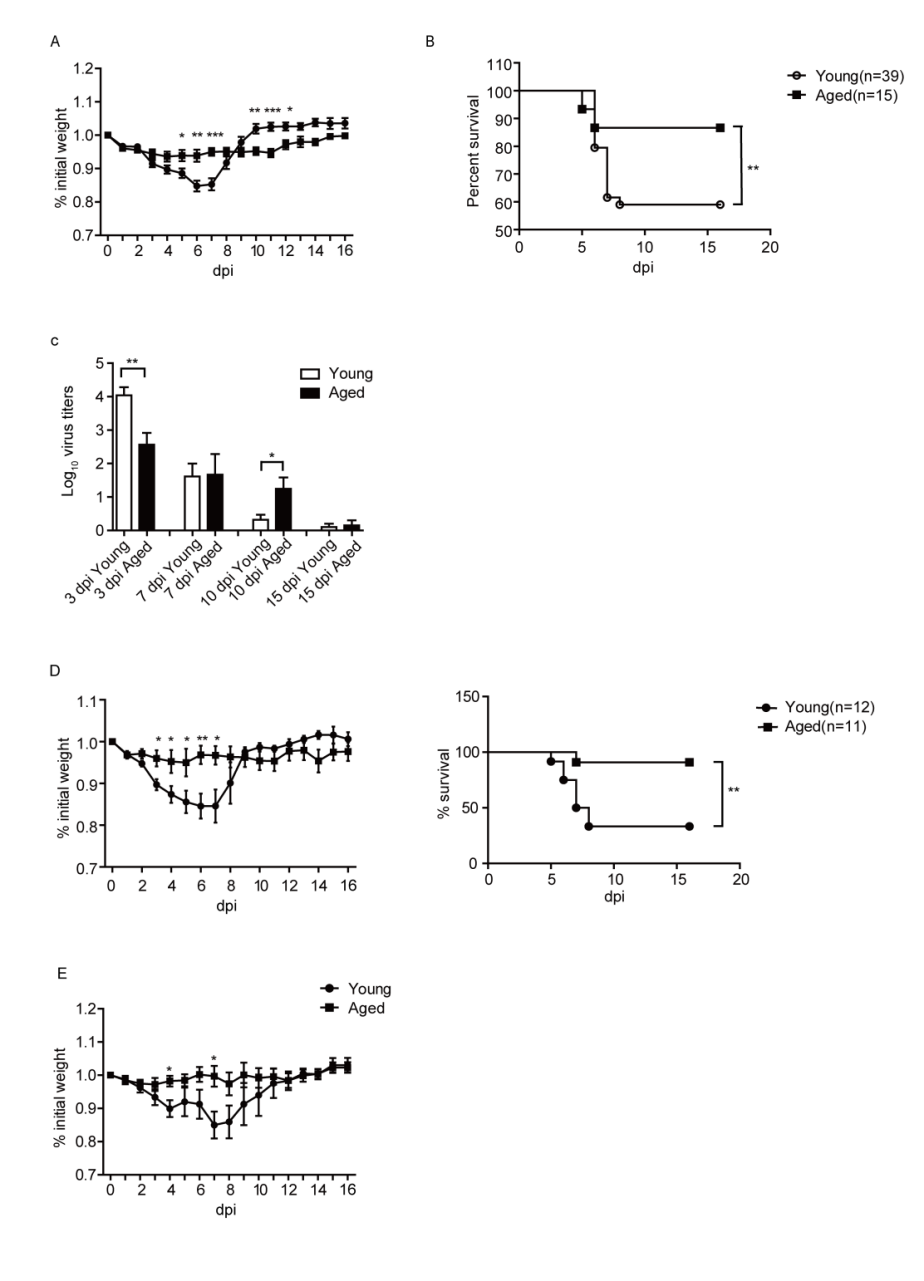
Young and aged mice exhibited different morbidity and mortality post PR8 infection. Young and aged B6 mice were infected i.n. with 10^3^ pfu of PR8 virus. Weight loss (A), survival (B) and viral titers (C) of infected mice were monitored. (D) weight loss and survival of the mice after infected with 10^4^ pfu of PR8 virus. (E) weight loss of the mice when the young mice were infected 10^3^ pfu of PR8 virus, while the aged mice were infected with 1.7×10^3^ PR8 virus. Data are from at least three independent experiments with more than 3 mice per group in each experiment. Data points indicate means±SD. *p<0.05,** *p*<0.01, *** *p*<0.001.

### Cytokine and chemokine analysis

Mice were sacrificed at the indicated times, and pulmonary homogenates were prepared. Supernatants were stored at -80°C. Cytokine and chemokine levels were measured using a Mouse Cytokine Magnetic 20-Plex Panel (Invitrogen) kit or a Mouse inflammation Panel Mix and Match Subpanel kit (BioLegend) according to the manufacturer’s instructions and read on a Luminex 100 (Bio-Rad) or a FACS LSRFortessa flow cytometer (BD Biosciences).

### Histopathology

For histological analysis, lung tissues were removed and fixed with 4 % paraformaldehyde for at least 12 h, dehydrated in a series of graded alcohols and embedded in paraffin. Tissue sections (5 mm) were cut and stained with HE. Histological sections were examined using a Zeiss Axio Imager M1 microscope equipped with an AxioCam HRc camera under control of AxioVision 4 software. The histological analysis was processed with Photoshop software.

### Statistical analysis

Statistical analysis was performed using Prism software (GraphPad). All statistical analyses were performed using an unpaired two-tailed Student’s t-test or two-way ANOVA test as applicable. When applicable, data are displayed as mean + SD.

## RESULTS

### Aged mice are more resistant to PR8 infection compared with young mice

To study the pathological reactions during influenza virus infection, young and aged C57BL/6 (B6) mice were intranasally (i.n.) infected with 10^3^ pfu PR8 virus. As shown in Fig. 1A, young mice lost weight rapidly after PR8 infection, lost more than 10% of body weights on 7 days post infection (dpi), and then gradually recovered to the normal level after 10 days. Infection also caused around 40% young mice died. Surprisingly, aged mice showed significantly reduced body weight loss and higher survival rate. Only around 10% of aged mice died after influenza virus infection (Fig. 1B).

We further investigated the virus titers in the lung of the young and aged mice at different time post infection. As shown in Fig. 1C, the virus titers were high in the young mice at 3 dpi, then decreased at 7 dpi. And at 10 dpi, viruses were largely cleared. While in the aged mice, the virus titer was significantly lower than the young mice at 3 dpi, and remained at a relatively higher level until 10 dpi. On 10 dpi, the virus titer in the lung of the aged mice was significantly higher than that of the young mice. After 10 dpi, the virus titer in the aged mice also decreased. At 15dpi, most of the viruses were cleared in both the aged mice and young mice. Overall, the virus loads in the lungs of the young mice were cleared faster than that of the aged mice. Next, we infected the young and aged mice with 10^4^ pfu of PR8, we observed the same trend that the aged mice exhibited a reduced weight loss and higher survival rate compared to the young mice (Fig. 1D).

Because the initial weight of the young and aged mice was different, the mean weight of the young mice was 19.5g, while the aged was 33.2g. To further investigate whether the increased survival of the aged mice correlated to their higher bodyweight, we infected the young mice with 10^3^ pfu PR8 virus, and infected the aged mice with 1.7×10^3^ pfu PR8 virus (the same fold of weight). As shown in Fig. 1E, even at the same dose per weight unit, aged mice lost less weight compared to young mice. Thus, these results indicated that the aged mice were more resistant than the young mice after PR8 infection.

### Overall reduced NK cell responses in the aged mice after infection

**Figure 2.**
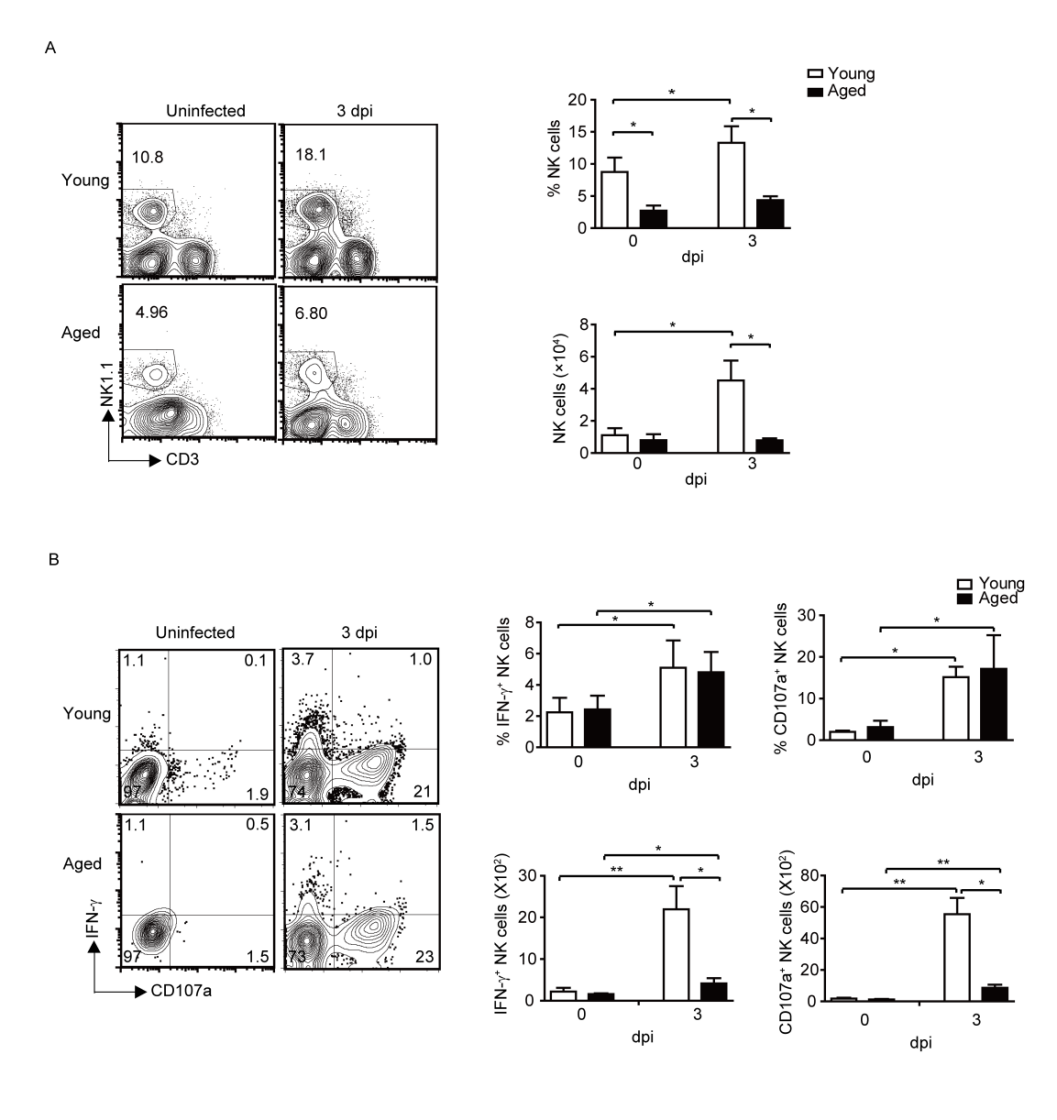
Overall reduced NK cell responses in the aged mice after PR8 infection. Young (n=39) and aged (n=15) B6 mice were infected i.n. with 1×10^3^ PFU PR8 virus. (A) The percentage and number of total NK cells in the lung of uninfected and infected young and aged mice. (B) The percentage and number of IFN-γ^+^ NK cells and CD107a^+^ NK cells in total NK cells. Data are representative of at least five similar experiments. Data points indicate means±SD. *p<0.05,** *p*<0.01.

Since NK cells are important effector cells in innate immune responses and shown to play very important roles in influenza virus infection [38], we first studied the infiltration and activation of NK cells in the lung of young and aged mice. As shown in Fig. 2A, aged mice had significantly lower percentage of NK cells in the lungs without infection. After infection, the percentage and total cell number of NK cells increased significantly in the young mice. Although the percentage of NK cells also showed a slightly increase in the aged mice after infection, however, the percentage and total cell number of NK cells in aged mice were significantly lower compared to that of young mice.

We further determined NK cell activation after infection. As shown in Fig. 2B, NK cells were activated after IAV infection, NK cells produced IFN-γ in both young and aged mice. NK cell surface CD107a expression level were also significantly increased in both young and aged mice after IAV infection. There were no significant differences in the percentage of IFN-γ^+^ or CD107a^+^ NK cells between young and aged mice after infection. However, because of the reduced number of IFN-γ^+^ or CD107a^+^ NK cells in the aged mice, the overall strength and magnitude of NK cell responses were decreased in the aged mice compared to that of young mice after IAV infection.

### The percentages of Neutrophils and DC cells are increased in the young mice but not in the aged mice after infection

**Figure 3.**
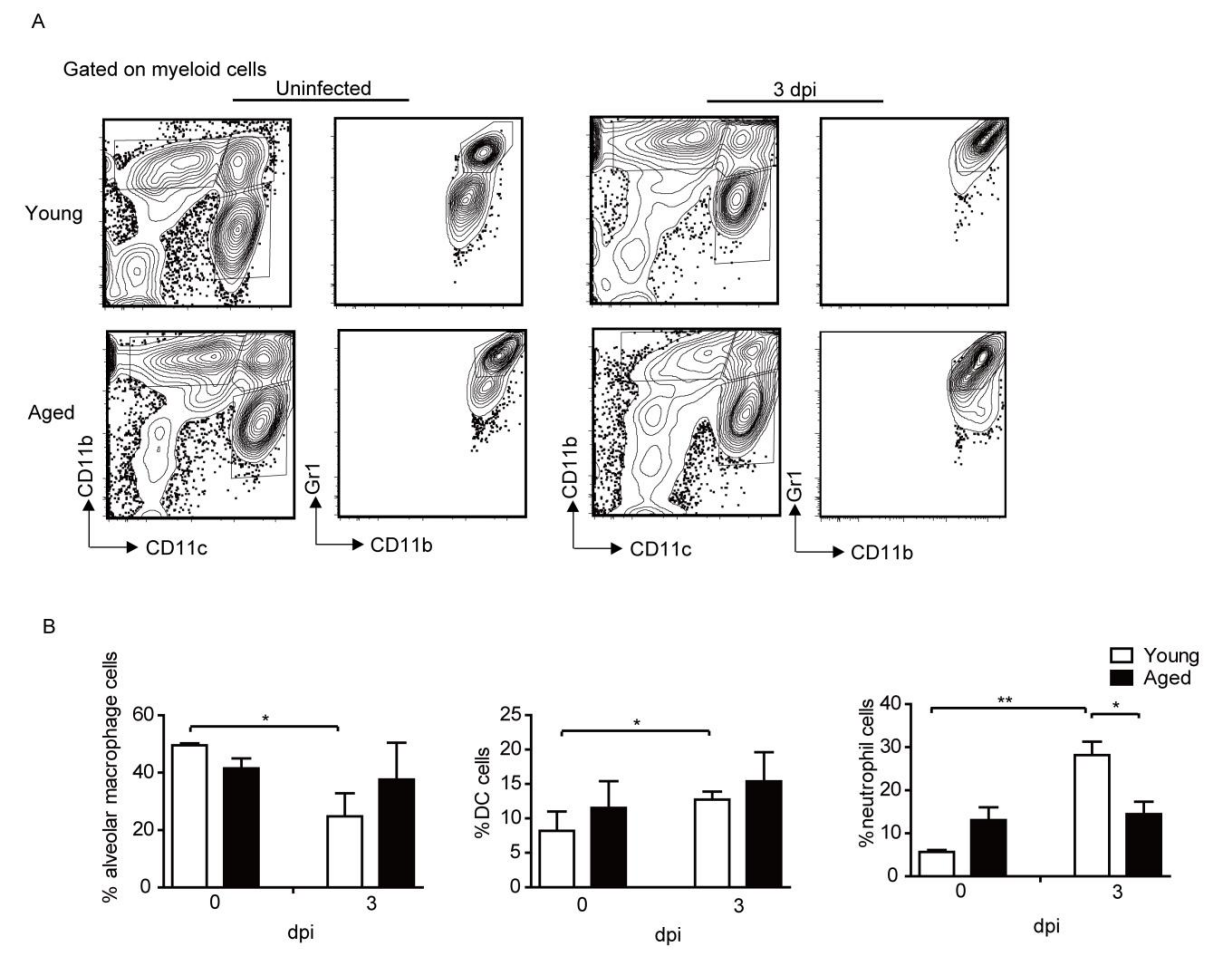
Neutrophils and DC cell percentage increased in the young mice but not in the aged mice after infection. Young and aged B6 mice were infected i.n. with 1×10^3^ PFU PR8 virus. (A) Gating strategy of cell populations. The cells were firstly gated on myeloid cells. Alveolar macrophages were CD11b^low/mid^CD11c^+^, we also confirmed that all the cells were F4/80^+^; DC cells were CD11b^high^CD11c^+^; neutrophils were CD11b^+^CD11c^-^Gr1^+^. (B) Percentage of the indicated cells before and after IAV infection. Data are representative of two similar experiments with 3-5 mice per group in each experiment. Data points indicate means ± SD. *p <0.05, ** *p*<0.01.

We further investigated the alveolar macrophages, neutrophils and DC cells in the lung of young and aged mice before and after infection. The gating strategy (Fig. 3A) of the cells was performed according to previous studies [36, 37]. Alveolar macrophages were gated on CD11b^low/mid^CD11c^+^ cells, we also confirmed that all the cells were F4/80^+^; DC cells were CD11b^high^CD11c^+^; neutrophils were CD11b^+^CD11c^-^Gr1^+^. As shown in Fig. 3B, the percentage of alveolar macrophages decreased in the lung of young mice after infection, which was consistent with a previous report [39]. While the percentage of neutrophils and DC cells was significantly increased in the young mice. However, the percentage of alveolar macrophages, neutrophils and DC cells all remained largely unchanged at 3 dpi in the lungs of the aged mice.

### Decreased and delayed kinetics of T cell responses in aged mice after infection

**Figure 4.**
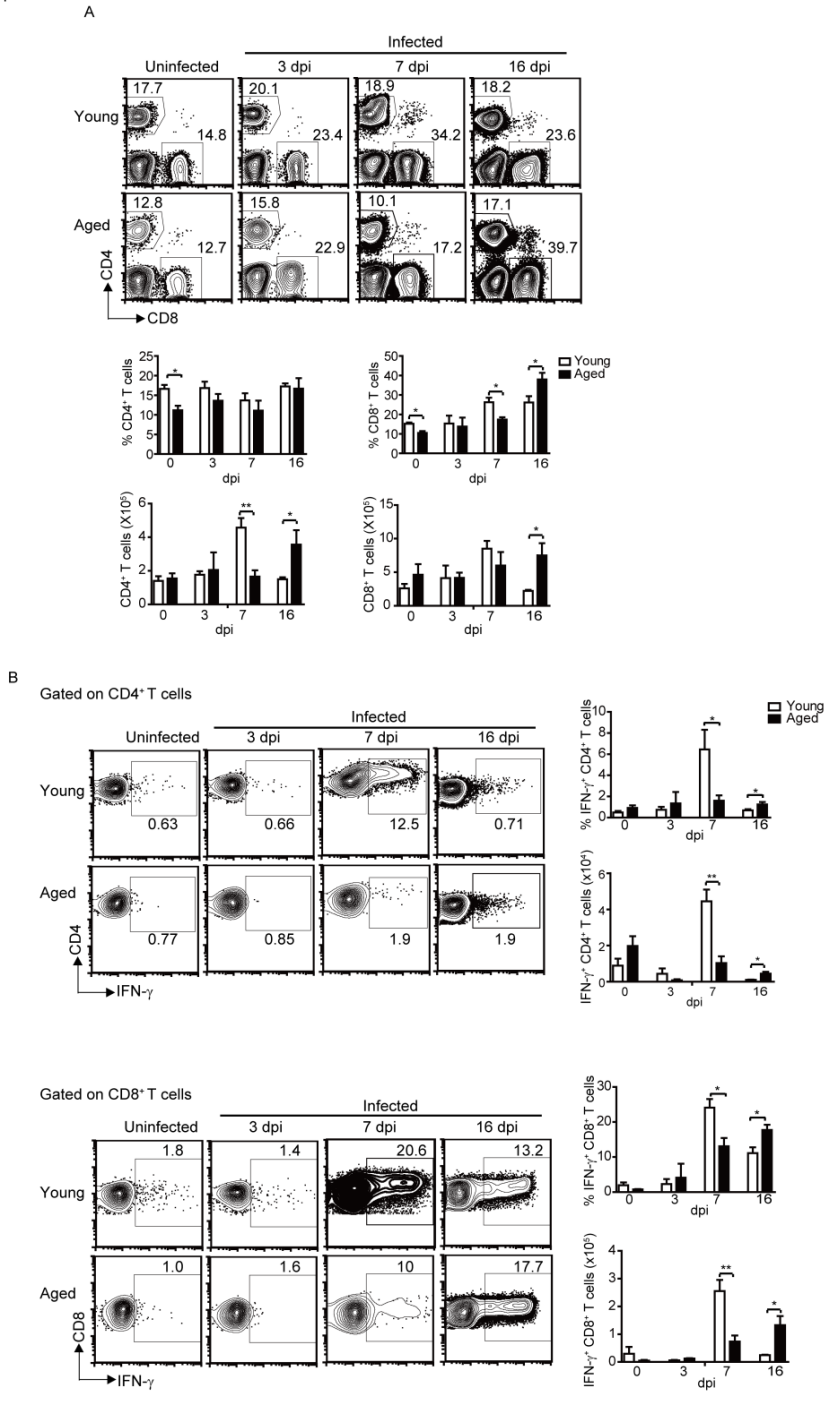
Delayed and reduced T cell responses in the aged mice after infection. Young and aged B6 mice were infected i.n. with 1×10^3^ PFU PR8 virus. (A) The percentage and the number of total CD4^+^ T and CD8^+^ T cells in the lung of young and aged mice at the indicated time points. (B) The percentage and the number of IFN-γ^+^ CD4^+^ T or CD8^+^ T cells in total CD4^+^ T or CD8^+^ T cells at the indicated time points, respectively. Data are representative of at least five similar experiments. Data points indicate means±SD. *p <0.05, ** *p*<0.01.

Next, we measured the percentage and activation of T cells in the lungs. As NK cells, the proportion of CD4^+^ T cells in aged mice was also significantly lower compared to the young mice without infection. After infection, CD4^+^ T cells percentage remained largely stable on 3, 7 and 16 dpi, and there was no significant differences between the aged mice and the young mice (Fig. 4A). But the total CD4^+^ T cells in the lung of young mice reached peak on 7 dpi, while in the aged mice, it reached peak on 16 dpi. Meanwhile, the CD4^+^ T cell responses in the young mice reached peaked on 7 dpi, with around 10% CD4^+^ T cells produced IFN-γ. After that, the percentage of activated CD4^+^ T cells went down. While in the aged mice, the CD4^+^ T cell responses were significantly lower compared to young mice on 7 dpi, but remained the similar level till 16 dpi, which was significantly higher compared to the young mice on 16 dpi (Fig. 4B). The number of the IFN-γ^+^CD4^+^ T cells showed a similar pattern.

**Figure 5.**
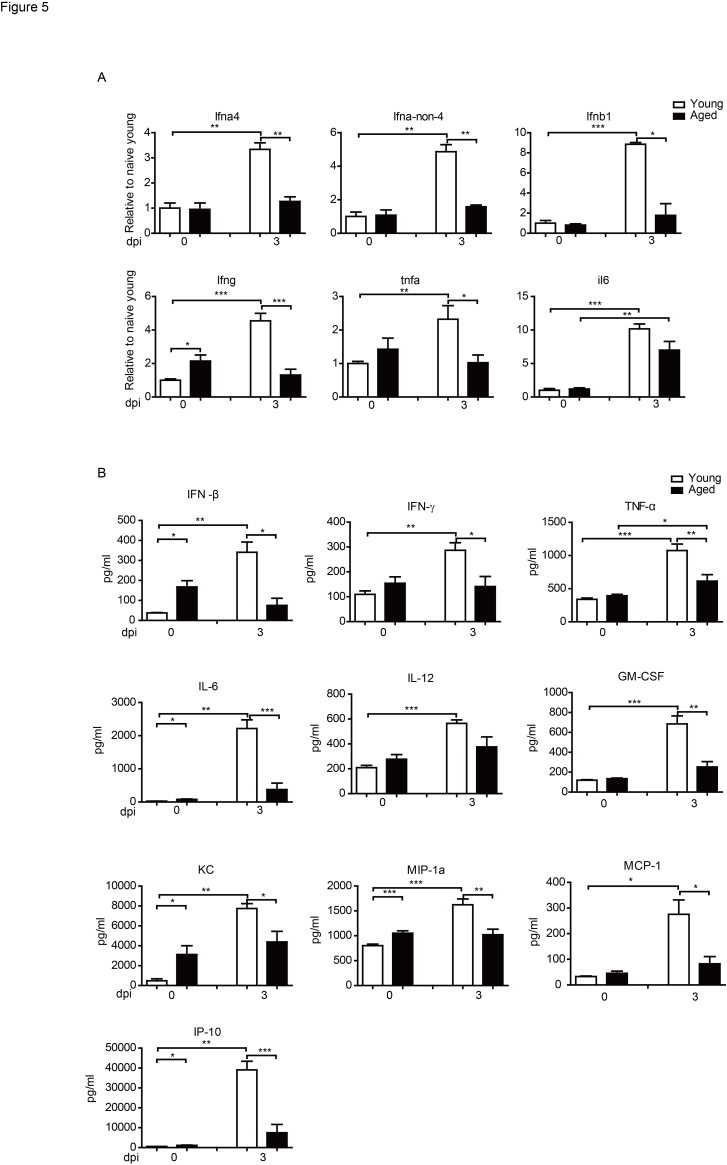
Reduced inflammation cytokines and chemokines in the aged mice after infection Young and aged B6 mice were infected i.n. with 1×10^3^ PFU PR8 virus. (A) The mice were sacrificed and pulmonary homogenates were prepared to measure the levels of the indicated molecules by real-time quantitative PCR at indicated times post infection. (B) The mice were sacrificed, and pulmonary homogenates were prepared to measure the levels of inflammatory cytokines and chemokines using a Luminex assay on 3 dpi. Data correspond to the mean±SD of six individual mice per group from two independent experiments. *p <0.05, **p <0.01, ***p < 0.001.

Compared to young mice, the percentage of CD8^+^ T cells in the lungs of aged mice also significantly reduced without infection. After infection, the CD8^+^ T cell in the lungs of young mice increased and reached peak on 7 dpi, then kept the similar level until 16 dpi. While CD8^+^ T cells in the aged mice showed a delayed increase, and reached a higher percentage on 16 dpi (Fig. 4A). The activation of CD8^+^ T cells also showed differences between young and aged mice. The percentage of IFN-γ^+^ CD8^+^ T cells reached peak on 7dpi in the young mice, after that, the percentage of activated CD8^+^ T cells went down. However, the CD8^+^ T cell responses showed a delayed and weaker kinetics in the aged mice. The percentage of IFN-γ^+^ CD8^+^ T cells increased slowly and reached peak on 16 dpi in the aged mice (Fig. 4B). The number of total CD8^+^ T cells and IFN-γ^+^CD8^+^ T cells had similar trend with the percentage.

Overall, there was significantly reduced CD4^+^ and CD8^+^ T cell responses in the aged mice On 7 dpi. While on 16 dpi, the CD4^+^ and CD8^+^ T cell responses were higher in the aged mice than that of the young mice. Therefore, the T cell responses in the lungs of the aged mice exhibited a delayed and decreased kinetics compared with the young mice.

### Reduced inflammation cytokines and chemokines in the aged mice after infection

Influenza virus infects the epithelial cells that line the respiratory tract [40]. Pro-inflammatory cytokines and chemokines activate and recruit leukocytes into the lungs and airways [41]. For example, MCP-1 induces the recruitment of NK cells and inflammatory monocytes that are important for the early control of virus replication. MIP-1α is important for the intense recruitment of neutrophils and contributes to the lung injury during IAV infection. IFN-α4 and IFN-β1 are necessary for the clearance of virus, and IFN-γ is important to regulate the cytokine production and CD4^+^ T cell activation during infection. TNF-α and IL-6 contribute to endothelium activation leading to expression of P-selectin, E-selectin, and integrins that are essential for leukocyte recruitment to the lungs [41-45].

Because the T cell responses in the aged mice exhibited a delayed and weaker kinetics after infection, we further checked the cytokine expression in lung after influenza virus infection. We first determined the cytokine levels in the young and aged mice by quantitative real-time PCR. As shown in Fig. 5A, at 3 dpi, transcripts of several cytokines, such as Ifna4, Ifna-non-4, Ifnb1, Ifng, Tfna, and Il6 increased significantly in the lung of the young mice. While in the aged mice, except Il6, transcripts of the other cytokines did not show significant increase after infection. We Further measured the cytokine levels using Luminex assays. As shown in Fig. 5B, PR8 infection in young mice resulted in significantly increased levels of cytokines in the lungs, including IFN-β, IFN-γ, TNF-α, IL-6, IL-12, and GM-CSF. But in aged mice, the cytokine levels were lower after infection. This was consistent with the results of Q-PCR.

Moreover, in the young mice, multiply chemokines, such as KC, MIP1-α, MCP-1, and IP-10 increased significantly in young mice on 3dpi, while there was no significant increase of those chemokines in aged mice. Thus, the early increase of multiply cytokines and chemokines in the young mice were likely to recruit more leukocytes infiltrating to the infected lung. These results were consistent with the faster and stronger T cell responses in the young mice compared to the aged mice.

### Delayed leukocytes infiltration and alleviated pulmonary pathology in the aged mice after PR8 infection

**Figure 6.**
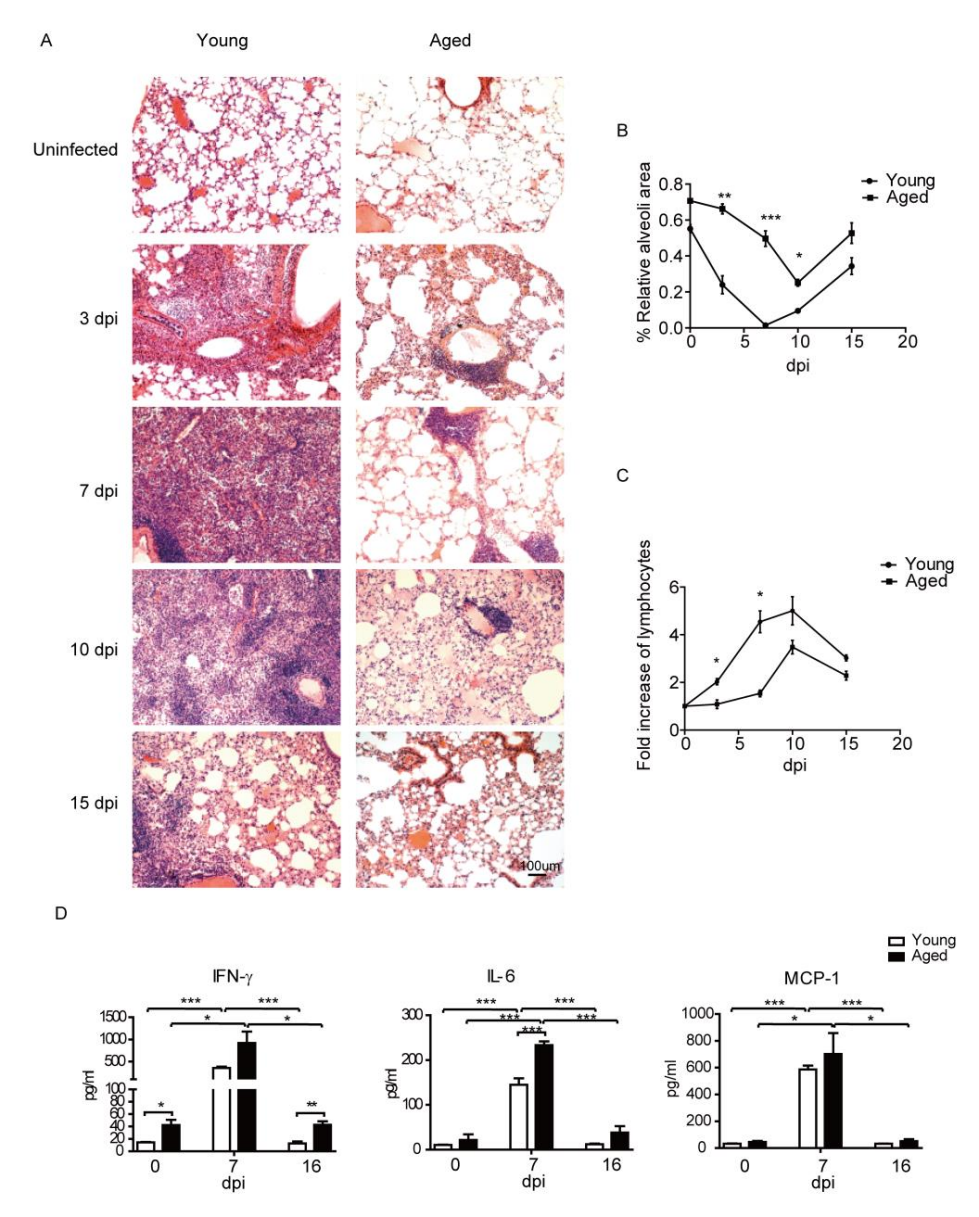
Reduced inflammation and lung damage in the aged mice after infection. Young and aged B6 mice were infected i.n. with 1×10^3^ PFU PR8 virus. (A) Hematoxylin-eosin staining (H&E) of the lung sectins in the young and aged mice at indicated time points. (original magnification×10, as labeled; scale bars, 100um). (B) Statistical analysis of relative alveoli area in lung. (C) Statistical analysis for fold of lymphocytes increase in lung, compared with uninfected mice. (D) Typical cytokine and chemokine levels at the indicated time. The mice were sacrificed at the indicated time, and pulmonary homogenates were prepared to measure the levels of inflammatory cytokines using a Legendplex kit. Data are representative of two experiments (two to three mice per group in each experiment). *p < 0.05, **p <0.01, ***p <0.001

The above data demonstrated reduced inflammation cytokines and delayed T cell responses in the aged mice after PR8 infection. We further performed histological analysis of the infected mice at different time to directly assess lung pathology. As shown in Fig. 6A, leukocytes infiltration was clearly seen on 3 dpi in young mice, while leukocytes of aged mice were only seen around some vessels. leukocytes infiltration reached peak in young mice on 7 dpi. In most severe areas, leukocytes were full of the lung tissue, almost no normal alveolar structures were found in the whole lung of the infected young mice. On the other hand, though clearly more leukocytes were found in the lungs of aged mice on 7 dpi, the cells were still around the vessel and did not move to the pulmonary parenchyma. On 10 dpi, leukocytes decreased and some alveoli were found in the lungs of young mice, while the leukocytes in the pulmonary parenchyma significantly increased in the lungs of aged mice, though some cells still gathered around the vessel. On 15 dpi, the lungs of young and aged mice both turned to recovery, with the leukocytes infiltration decreased and the alveolar structure partially restored.

The above histological analysis indicated that during PR8 infection, the aged mice showed a delayed and reduced leukocytes infiltration, and alleviated pulmonary inflammation and pathology. The relative alveoli area in the lungs of aged mice was significantly higher than that of the young mice on 3, 7 and 10 dpi (Fig. 6B). Further, the fold of leukocytes increase in the aged mice was lower than that of the young mice at all the time after PR8 infection, and reached statistical significance on 3 and 7 dpi (Fig. 6C). In addition, we further detected some typical cytokine and chemokine levels at later time points after infection. As shown in Fig. 6D, different with 3 dpi, IFN-γ, IL-6, and MCP-1 levels were significantly increased in the aged mice on 7 dpi compared to uninfected mice. IL-6 level was significantly higher in the aged mice compared to that of the young mice on 7 dpi, while IFN-γ and MCP-1 levels were at the same range with the young mice. On 16 dpi, the levels of cytokine and chemokine were all decreased in young and aged mice, however, the IFN-γ level was significantly higher in the aged mice compared to that of the young mice, which was consistent with the late onset of T cells responses in the aged mice.

All together, histological analysis revealed that delayed and decreased lymphocytes infiltration and alleviated lung pathology in the aged mice after PR8 infection. Furthermore, the delayed onset of cytokines and chemokines in the aged mice were also consistent with the delayed onset of T cell responses.

## DISCUSSION

In this study, we demonstrated that when challenged intranasally with IAV PR8 virus, the aged mice were more resistant than the young mice. The reduced inflammation cytokines and lower strength of NK and T cell immune responses in the aged mice might contribute to the alleviated lung pathology and increased survival rate after PR8 infection.

In humans and other mammals, the initial protective response to influenza virus deposition on the mucosa of the respiratory tract is both physiological and immunological. Innate immune cells, such as natural killer (NK) cells, alveolar macrophages, and dendritic cells (DCs) play critical roles not only in the initial control of viral replication but also in the regulation of influenza virus-specific adaptive immunity [46]. The cytokines and chemokines produced by these cells are important defense against IAV infection preventing the replication and spread of the virus. However, the nonspecific innate immune response can be locally destructive if not appropriately contained [41]. Certain influenza viruses, such as highly pathogenic avian influenza (HPAI) A/H5N1 strains, can trigger an overly inflammatory innate immune response, often called “cytokine storm” [47]. Therefore, the inflammatory response induced by the IAV infection functions as a double-edged sword, it is necessary to protect against viral infection but may also cause severe pulmonary injury.

Previous studies have shown that increased survival in the IL-15^-/-^ mice or NK cell-depleted wild-type mice after IAV infection was associated with significantly lower lung lesions, decreased mononuclear cells and neutrophils in the airway lumen as well as lower proinflammatory cytokines, including IL-6 and IL-12 in the bronchoalveolar lavage fluid [48]. Our results showed that the early inflammatory cytokines, lung pathology, neutrophils infilitration, and the overall NK cell responses in the lungs of aged mice were significantly lower than the young mice on 3 dpi, which was consistent with the reduced bodyweight loss in the aged mice after PR8 infection. Therefore, the higher overall NK cell responses in the early infection may contribute to the enhanced lung pathology in the young mice. Further, NF-kB activation is a hallmark of inflammatory and antiviral responses mediated especially by type I interferons that block viral replication, however, previous studies showed that active NF-kB signaling is required for effective influenza virus infection [49-51]. Thus, the higher viral loads in the lungs of the young mice on 3dpi were in accord with the early activation of inflammation and lung injury. Although the early viral loads were high in the young mice, the virus were cleared rapidly with the effective and strong T cell responses, as most of the viruses were cleared on 10 dpi. On the other hand, in the aged mice, even though the viral load was lower on 3 dpi compared to the young mice, the viral clearance were prolonged because of the delayed and milder T cell responses. Of note, even the peak strength of CD4^+^ T cell responses were severely reduced in the aged mice (1.593±0.5105 in the aged mice on 7 dpi compared to 6.446±1.863 in the young mice on 7 dpi), the strength of peak CD8^+^ T cell responses in the aged mice (16 dpi) were at a similar level, albeit a little reduced, compared to that in the young mice on 7 dpi (17.30±0.9212 in the aged mice on 16 dpi compared to 24.77±2.117 in the young mice on 7 dpi). Therefore, the delayed and milder T cell responses in the aged mice were still capable to finally clear the virus, as the viral loads in the aged mice were mostly cleared on 15 dpi. On the other side, CD8^+^ T cells are partly responsible for the pathogenesis of acute pneumonia caused by influenza A virus [52]. Thus, the lower CD8^+^ T cell responses in the aged mice might have also contributed to the reduced lung lesions in the aged mice. However, many factors might influence the final outcome after IAV infection.

Due to the double-edged sword effect of the inflammatory response, mounting an effective immune response, while also protecting tissue integrity, is critical for host survival [53]. Previous studies indicated that the immune response to pathogen infection differ by age [54, 55]. The mice genetically resistant to mousepox lose complete resistance at mid-age [56]. In the H5N1 and 2009 pandemic H1N1 infection, most of the hospitalized patients were young people with median aged at 25. While in the H7N9 infection, the median age of patients was 63 [31]. Those reports indicate that different influenza viruses might have distinct age patterns of severe infection and symptom.

Healthy aged individuals have a significantly higher basal inflammatory state where circulating levels of cytokines, including IL-6, TNF-α and IL-1β, are elevated [10]. This progressive pro-inflammatory state, termed “inflamm-aging,” affects the phenotype/function of cells present in the aged, including monocytes, macrophages, and DC cells [57]. Previous study reported that the number of neutrophils was relatively higher in the elderly, but the effector functions such as chemotaxis and intracellular killing were decreased. And the macrophages and DC cells also showed age-related changes, including the decreased cytotoxicity, intracellular killing and antigen presentation. These changes resulted in the impaired adaptive immune responses in the elderly [54, 55, 58]. In our research, the alveolar macrophages decreased in the lung of young mice on 3 dpi, which was consistent with a previous report that more than 90% of resident alveolar macrophages were deleted in the first week after influenza infection [39]. While the percentage of DC and neutrophils increased in the lungs of the young mice. However, alveolar macrophages, neutrophils and DC cells remained largely unchanged in the lungs of the aged mice on 3 dpi.

In our research, the aged mice we used were 16-19 months. According to their life span, those mice were at their early aging stage. The strong immune response in the young mice resulted in faster clearance of IAV virus, but also caused severe lung injury, which led to higher mortality and pronounced bodyweight loss. While in the aged mice, the delayed and weaker immune responses were still capable to eventually clear the virus infection but with limited tissue damage. Therefore, the aged mice exhibited a more resistant phenotype during the IAV infection. Previous paper reported when infected with sub lethal doses of (50-100pfu) PR8,compared to adult mice, aged mice (18-19M, BALB/C) had higher morbidity [59]. However, some research also investigated that aged mice (2.5-years-old, C57BL/6) were more resistant to the PR8 infection than the young mice [60]. Therefore, the resistance of the young and aged mice to influenza virus may be influenced by the age, physical status and strain of the mice, and infecting virus doses.

Further, in our study, we only investigated the primary responses to the IAV infection. Pre-existing cross-reactive anti-influenza immunity or second bacterial infections is not a mechanism to be considered for the age-related phenotype in our research. We only investigated the primary infection of influenza virus, and in this situation, the aged mice were more resistant than the young mice. But in the clinic, many factors might contribute to severe outcome after IAV infection, such as secondary opportunistic bacterial infections [61]. Moreover, our results support the hypothesis that the "cytokine storm" is one of the main cases of the high mortality rates in highly pathologic viral infections, such as SARS and H5N1 infections, in the young people rather than the aged population [23, 25].

**Figure 7.**
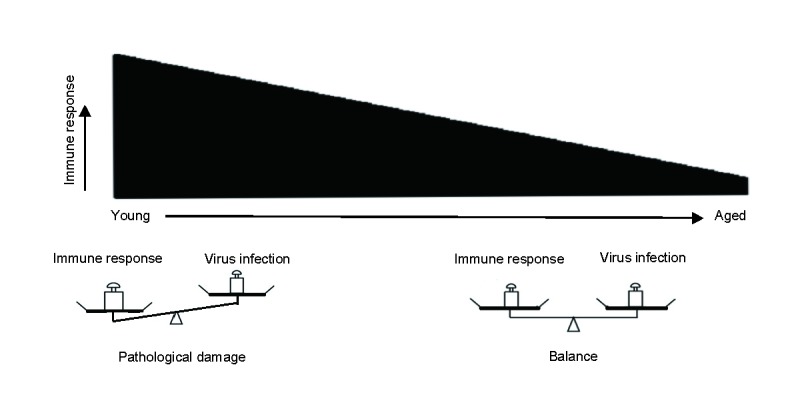
Model of the strength of immune responses and pathological tissue damage. The strength of immune responses decreases with age. In the young mice, the excessive immune responses result in severe pathological damage. In the aged mice, the milder immune response might balance viral clearance and tissue integrity, thus exhibiting more resistant phenotype.

Aging is a continuum of changes that are progressive with time. The decline of immune functions with ageing might also be a gradual and progressive process. Therefore, from young to ageing, there is a gradual decline in the strength and speed of the host immune responses against viral infections. For some highly pathogenic viral infections, the excessively strong immune responses initiated in the young host might be harmfully for the host because of severe pathological damage. On the other hand, low strength of immune responses in the aged host might result in viral persistence. However, during the whole continuum changes, there might be a balance point where the moderate immune responses are still capable to clear the infection while with reduced pathologic damage, which are beneficial for the host. This model is illustrated in Fig. 7. This balance point might vary with different virus, infecting dose and route.

In summary, our data demonstrates that the moderate immune responses as a decline with ageing balance the immune pathology and viral clearance, providing survival advantage for the aged mice during IAV infection. Our results provide important insight to our basic knowledge of immunosenescence and immune defenses to invading pathogens. Further, our results indicate that age factors should be considered when investigating the vaccination and therapeutic strategies for severe IAV infection.
